# Geographical and environmental determinants of the genetic structure of wild barley in southeastern Anatolia

**DOI:** 10.1371/journal.pone.0192386

**Published:** 2018-02-08

**Authors:** Reza Pournosrat, Selma Kaya, Salar Shaaf, Benjamin Kilian, Hakan Ozkan

**Affiliations:** 1 Department of Agronomy and Plant Breeding, College of Agriculture and Natural Resources, Sanandaj Branch, Islamic Azad University, Sanandaj, Iran; 2 University of Çukurova, Faculty of Agriculture, Department of Field Crops, Adana, Turkey; 3 Leibniz Institute of Plant Genetics and Crop Plant Research (IPK) Gatersleben, Genebank Department, Genome Diversity Group, Seeland, Germany; Ben-Gurion University, ISRAEL

## Abstract

Despite the global value of barley, compared to its wild progenitor, genetic variation in this crop has been drastically reduced due to the process of domestication, selection and improvement. In the medium term, this will negatively impact both the vulnerability and yield stability of barley against biotic and abiotic stresses under climate change. Returning to the crop wild relatives (CWR) as sources of new and beneficial alleles is a clear option for enhancing the resilience of diversity and adaptation to climate change. Southeastern Anatolia constitutes an important part of the natural distribution of wild barley in the Fertile Crescent where important crops were initially domesticated. In this study, we investigated genetic diversity in a comprehensive collection of 281 geo-referenced wild barley individuals from 92 collection sites with sample sizes ranging from 1 to 9 individuals per site, collected from southeastern Anatolia and 131 domesticated genotypes from 49 different countries using 40 EST-SSR markers. A total of 375 alleles were detected across entire collection, of which 283 were carried by domesticated genotypes and 316 alleles were present in the wild gene pool. The number of unique alleles in the wild and in the domesticated gene pool was 92 and 59, respectively. The population structure at K = 3 suggested two groups of wild barley namely G1-W consisting wild barley genotypes from the western part and G1-E comprising those mostly from the eastern part of the study area, with a sharp separation from the domesticated gene pool. The geographic and climatic factors jointly showed significant effects on the distribution of wild barley. Using a Latent Factor Mixed Model, we identified four candidate loci potentially involved in adaptation of wild barley to three environmental factors: temperature seasonality, mean temperature of driest quarter, and precipitation of coldest quarter. These loci are probably the targets of genomic regions, with potential roles against abiotic stresses.

## Introduction

Archeological evidence suggests that barley (*Hordeum vulgare* L. ssp. *vulgare*) was domesticated more than 10,000 years ago in the Fertile Crescent [1]. Today it is extensively being cultivated in different regions of the world and used for animal feed, brewing malts, and human consumption. Barley was selected from its wild progenitor *H*. *vulgare* L. ssp. *spontaneum*. Both taxa are diploid (2*n* = 14), predominantly self-pollinated and fully interfertile [1]. The core distribution area of wild barley is the Fertile Crescent, but it naturally occurs from western North Africa to the Himalayas [2]. Compared to their wild relatives, the domestication process of crop plants usually leads to genetic bottlenecks causing considerable reduction of genetic variation, which is a major concern for plant breeders today. This reduction of diversity has been shown to be severe in barley reaching approximately 50% [3]. Therefore, identifying new resources to increase variation in barley is essential to reduce the vulnerability of new varieties to various biotic and abiotic stresses under changing climates. One approach is to identify useful alleles within crop wild relatives and landrace genepools [4]. To achieve this goal, the analysis of genetic variation of crop plants and their wild progenitors is important, considering materials from the center of domestication and/or the center of highest diversity [5]. However, in this process, knowledge of geographical distribution of genetic diversity of plants is crucial for collecting, protecting, and monitoring genetic resources [6]. In addition, genetic diversity is influenced by both geographical distribution; i.e., isolation by distance (IBD), and by environmental conditions; i.e., isolation by environment (IBE) [7]. Therefore, ecological and geographical data have been considered as important factors to improve sampling strategies and managing genetic diversity [8,9].

In the process of IBD, geographical distances confine the gene flow. It is expected that genetic differentiation will increase with increased physical distances between populations [10]. On the other hand, during the IBE process, natural selection affects genetic diversity and genetic differentiations among adjacent populations through effects of environmental factors [10,11]. Geographical factors can affect genetic structure on a large spatial scale, while ecological and environmental factors affect this parameter across both space and time [12,13]. Recent studies suggest the relative contribution of IBD and IBE in deriving genetic diversity at the species level, with a few researchers showing the relative significance of IBD, IBE, and historical climatic changes on genetic diversity [14–16]. Since 1970, genetic diversity of wild barley has been extensively investigated using morphological traits, isozymes, and molecular markers from different eco-geographical regions [16–26]. Previous studies indicated that genetic variation of wild barley is attributed to both physical distance [6] and to environmental gradients [18,26]. Geographical studies, on both micro and macro scales, have shown that footprints of evolutionary forces could be detected by exploiting genetic markers [27,28]. Microsatellite markers (Simple Sequence Repeats markers, SSRs) are extensively used exploited in population and landscape genetics. In fact, they are able to provide lucrative information about population differentiation and their structure [29,30]. The most prominent advantages of SSRs are their high repetitiveness and high polymorphism. SSRs are multi allelic and co-dominant [30]. As part of the Fertile Crescent, the southern areas of Turkey has been considered as one of the primary origins of wild barley [18]. It has been suggested that agriculture have started, probably independently, in at least two areas: one in the southeastern Anatolia and the Southern Levant [31]. Recently, two genes (*btr1 and btr2*) have been identified as responsible for non-brittle rachis in domesticating barley. The *btr1*-type barley emerged in the Southern Levant prior to the appearance of *btr2*-types in the Northern Levant [32]. More recently, a novel mutation conferring to non-brittle rachis at *Btr1* gene called *btr1b* has been reported to be and has been referred to wild accessions from Gaziantep region in Turkey [33]. This can further reflect the importance of southeastern Anatolia for genetic diversity of wild barley.

In this study we utilized EST-SSR markers to investigate the patterns of genetic diversity of collected the largest wild barley sample collected to date from southeastern Anatolia. In addition, we incorporated a worldwide collection of diverse landraces and cultivars mainly maintained in gene banks to compare diversity between the two different gene pools. The major objectives of the study were: a) to investigate the genetic structure and diversity of wild barley in Turkey and compare it with the domesticated varieties worldwide, b) to examine the relative importance of geographical and environmental factors on the patterns of genetic variation in wild barley; and c) to identify the potentially most important eco-geographical variables associated with forming the genetic makeup and variation of barley in southeastern part of Turkey.

## Materials and methods

### Plant materials, DNA extraction, and EST-SSR genotyping

**Fig 1:**
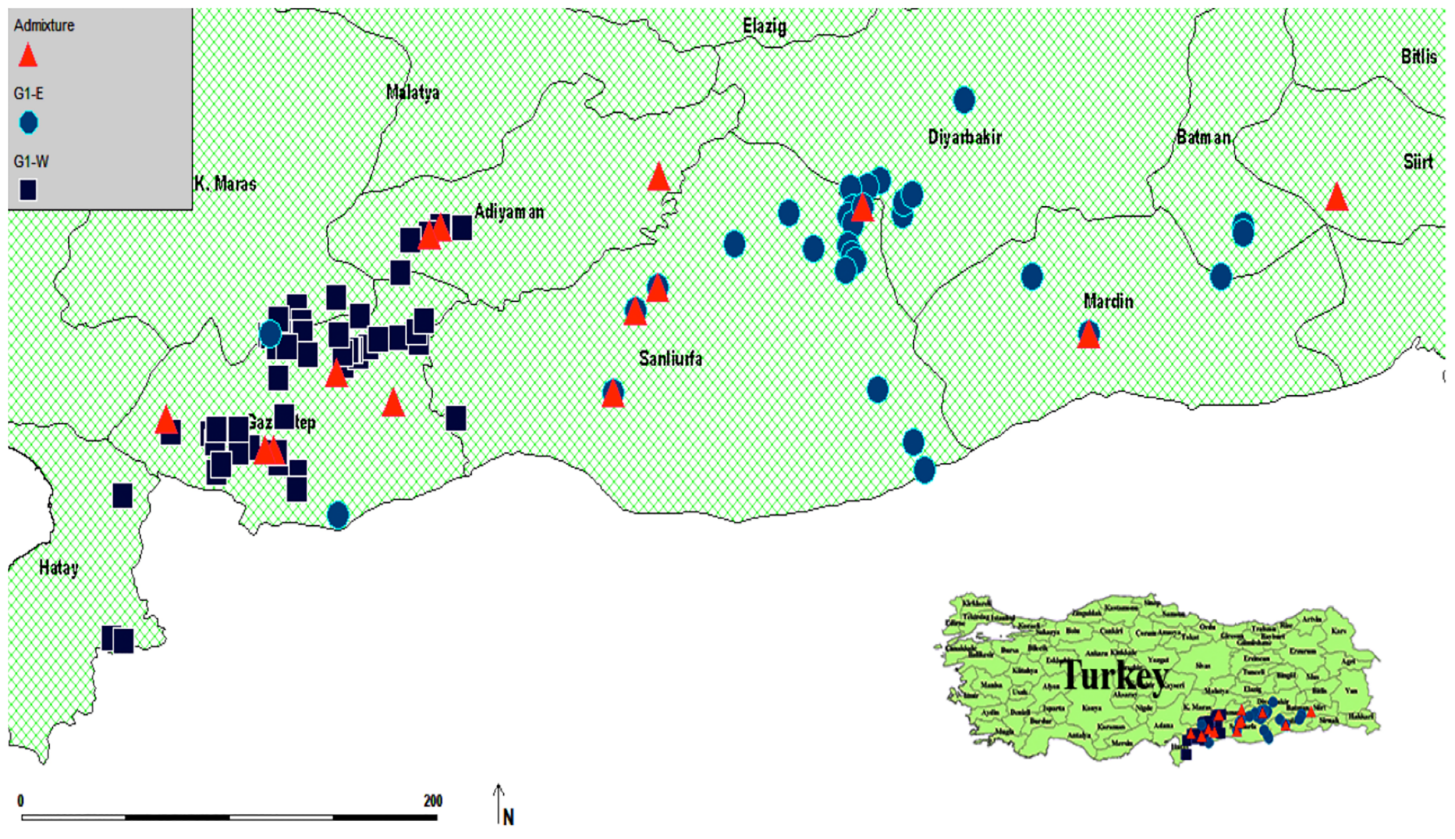
Geographical distribution of wild barley samples across the collection sites in Turkey. G1-E and G1-W are indicated by light blue circles and dark blue squares, respectively, showing the wild barley populations belonging to two groups inferred by STRUCTURE at K = 2 with a membership coefficient of ≥70%. Admixtures are shown using red triangles.

Wild barley individuals were freshly collected from 72 collecting sites from southeastern Anatolia. Whenever possible, we included additional wild barley individuals from *ex situ* repositories to increase the sample size and to fill collection gaps in the study area. This lead to a total number of 92 collection sites with sample sizes ranging from 1 to 9 individuals per site. The collection was further enriched with a diverse set of domesticated genotypes. A total of 281 wild and 131 domesticated genotypes (landraces and cultivars) genotypes were selected for the study. Geographic data including longitude, latitude, and altitude were recorded using global positioning system (GPS) to obtain the sampling location of wild barley (Fig 1, S1 Table).

Prior to genotyping all accessions were genetically purified by single seed descent (SSD). Four seeds from individual spikes per genotype were sown under greenhouse conditions. After three weeks, two fresh leaves were used for DNA extraction. Forty-five fluorescence-labeled EST-SSR markers covering all seven chromosomes of the barley genome, were selected for the study [17,34] (S2 Table). PCR reactions were performed based on the protocol described in previous studies [17,35]. The PCR amplification products were separated on a MegaBACE 1000 Capillary sequencer (Amersham Biosiences). The fragment sizes were analyzed by MegaBACE Fragment Profiler software version 1.2. After subsequent manual inspection, a total of 40 markers with missing values of less than 0.1 were selected for all analyses reported in the following sections (Table 1).

**Table 1:** Marker information and diversity statistics analyzed separately for the whole collection and for the wild and domesticated barley separately. Chr: Chromosome; FR: Fragment range; NA: Number of alleles; MAF: Major allele frequency; PIC: Polymorphism information content; GD: Gene diversity; D: Domesticated; W: Wild; All: All accessions.

		FR			NA			MAF			PIC			GD		
Marker	Chr	W	D	All	W	D	All	W	D	All	W	D	All	W	D	All
**GBM-1002**	1H	261–285	261–**363**	261–**363**	9	9	14	0.525	0.711	0.362	0.622	0.409	0.753	0.659	0.452	0.781
**GBM-1007**	1H	184–228	196–218	184–228	**20**	10	20	**0.139**	0.372	0.214	**0.904**	0.672	0.881	**0.907**	0.716	0.890
**GBM-1013**	1H	162–174	165–174	162–174	5	4	5	0.768	0.744	0.589	0.312	0.357	0515	0.365	0.404	0.573
**GBM-1029**	1H	224–232	224–230	224–232	5	4	5	0.674	0.321	0.552	0.434	0.688	0.561	0.487	0.737	0.612
**GBM-1061**	1H	324–354	315–354	315–354	9	9	11	0.455	0.435	0.395	0.685	0.682	0.756	0.720	0.720	0.779
**GBM-1334**	1H	117–135	117–138	117–138	6	5	8	0.462	0.727	0.484	0.552	0.351	0.521	0.627	0.413	0.601
**GBM-1461**	1H	186–230	184–234	184–234	19	16	**24**	0.242	**0.229**	0.174	0.886	0.819	**0.908**	0.893	0.839	**0.914**
**GBM-1035**	2H	274–282	272–282	272–282	5	6	6	0.604	0.425	0.496	0.517	0.676	0.622	0.568	0.718	0.668
**GBM-1047**	2H	207–219	210–219	207–219	5	4	5	0.430	0.366	0.383	0.563	0.595	0.624	0.637	0.667	0.682
**GBM-1063**	2H	196–220	196–220	196–220	7	7	7	0.437	0.471	0.447	0.681	0.678	0.687	0.719	0.711	0.722
**GBM-1208**	2H	142–156	138–164	138–164	8	9	10	0.358	0.626	0.390	0.726	0.499	0.726	0.761	0.547	0.757
**GBM-1218**	2H	134–144	130–150	130–150	4	7	8	**0.917**	0.430	0.654	**0.151**	0.596	0.487	**0.156**	0.658	0.527
**GBM-1459**	2H	158–168	158–174	158–174	6	9	9	0.639	0.511	0.477	0.449	0.656	0.630	0.514	0.685	0.678
**GBM-1031**	3H	284–292	284–296	284–296	5	7	7	0.732	0.359	0.613	0.351	0.714	0.554	0.410	0.753	0.579
**GBM-1110**	3H	225–237	210–237	210–237	5	6	6	0.468	0.492	0.429	0.603	0.590	0.639	0.659	0.647	0.691
**GBM-1280**	3H	279–300	279–303	279–303	8	9	9	0.576	0.409	0.484	0.600	0.695	0.660	0.628	0.732	0.695
**GBM-1405**	3H	268–304	276–304	268–304	6	8	9	0.352	0.336	0.294	0.676	0.724	0.737	0.725	0.762	0.773
**GBM-1413**	3H	155–170	150–170	150–170	4	5	5	0.568	0.429	0.490	0.456	0.597	0.558	0.543	0.664	0.627
**GBM-1003**	4H	189–225	189–210	189–225	10	8	11	0.196	0.616	0.328	0.874	0.524	0.802	0.862	0.566	0.821
**GBM-1015**	4H	194–270	186–274	186–274	**20**	**20**	22	0.208	0.240	0.217	0.874	0.859	0.887	0.884	0.871	0.894
**GBM-1018**	4H	252–270	258–267	252–270	7	4	7	0.352	0.424	0.356	0.703	0.601	0.736	0.743	0.668	0.768
**GBM-1020**	4H	238–250	244–250	238–250	5	4	5	0.675	0.433	0.597	0.360	0.591	0.464	0.448	0.659	0.539
**GBM-1221**	4H	106–148	106–132	106–148	16	8	17	0.397	0.504	0.297	0.787	0.627	0.838	0.801	0.669	0.851
**GBM-1323**	4H	111–165	114–153	111–165	15	7	17	0.426	0.446	0.314	0.673	0.703	0.780	0.714	0.733	0.802
**GBM-1501**	4H	268–288	268–284	268–288	6	5	6	0.736	0.665	0.713	0.374	0.447	0.401	0.418	0.499	0.447
**GBM-1026**	5H	210–216	208–216	208–216	4	5	5	0.430	0.541	0.442	0.574	0.513	0.563	0.642	0.587	0.634
**GBM-1064**	5H	284–300	284–300	284–300	5	5	5	0.702	0.764	0.497	0.446	0.397	0.612	0.477	0.422	0.661
**GBM-1176**	5H	280–298	280–296	280–298	8	9	10	0.388	0.259	0.350	0.701	0.779	0.756	0.739	0.807	0.748
**GBM-1363**	5H	111–150	117–150	111–150	6	4	6	0.495	0.511	0.448	0.500	0.397	0.473	0586	0.514	0.567
**GBM-1483**	5H	126–174	168–171	126–174	11	**2**	11	0.818	0.812	0.816	0.299	0.259	**0.290**	0.317	0.306	0.315
**GBM-1008**	6H	165–189	159–183	159–189	8	7	10	0.270	0.457	0.330	0.779	0.642	0.759	0.807	0.690	0.788
**GBM-1075**	6H	294–304	290–314	290–314	4	7	7	0.718	0.550	0.707	0.333	0.541	0.420	0.364	0.602	0.460
**GBM-1212**	6H	96–111	99–111	96–111	6	5	6	0.539	0.443	0.421	0.461	0.659	0.625	0.553	0.704	0.682
**GBM-1256**	6H	344–**360**	334–356	334–360	8	6	8	0.494	0.664	0.549	0.527	0.488	0.548	0.602	0.521	0.605
**GBM-1404**	6H	262–270	258–270	258–270	**3**	4	**4**	0.830	**0.945**	0.866	0.267	**0.103**	**0.222**	0.293	**0.105**	**0.238**
**GBM-1033**	7H	270–286	270–292	270–292	8	8	9	0.821	0.504	0.721	0.312	0.556	0.440	0.320	0.622	0.461
**GBM-1060**	7H	207–213	207–216	207–216	**3**	4	**4**	0.737	0.584	0.688	0.324	0.426	0.362	0.393	0.521	0.442
**GBM-1419**	7H	**90**–135	**85**–135	**85**–135	7	10	11	0.482	0.695	0.334	0.667	0.484	0.718	0.701	0.501	0.754
**GBM-1464**	7H	124–172	142–218	128–218	11	7	16	0.594	0.330	0.447	0.585	0.727	0.745	0.611	0.763	0.766
**GBM-1516**	7H	**90**–108	90–108	90–108	9	10	10	0.664	0.517	0.477	0489	0.593	0.679	0.522	0.642	0.710
**Overall**	**----**	**----**	**----**	**----**	**316**	**283**	**375**	**----**	**--**	**----**	**----**	**----**	**----**	**----**	**----**	**----**
**Mean**	----	----	----	----	**7.9**	**7.0**	**9.4**	**0.535**	**0.507**	**0.437**	**0.551**	**0.573**	**0.623**	**0.594**	**0.620**	**0.660**

### Data analysis

Genetic diversity parameters including the number of alleles per each locus, observed heterozygosity, gene diversity, polymorphism information content (PIC), and major allele frequency (MAF) were calculated using software Power Marker software version 3.25 [36]. PIC value is an indicator of the probability of finding polymorphism between two random samples in germplasm. This statistic is widely used to explain the amount of genetic diversity that could be related to single nucleotide changes or insertions/deletions [37].

### Analysis of population structure

The population structure was investigated at three levels (L): across the entire population of domesticated and wild barley (L1), wild and domesticated barley from Turkey (L2), and wild barley only (L3). To infer genetic structure of the population, a Bayesian clustering approach was used using STRUCTURE software version 2.3.4, which assigns individuals to pre-defined clusters (K) based on their membership of a cluster [38,39]. The number of K was set to vary from 1 to 20 with 10 independent simulations per each K. For each run we used an admixture model and also the correlated allele frequencies option. The initial burn-in length was set to 100000 followed by 100000 Markov Chain Monte Carlo (MCMC) iterations. To identify the appropriate number of K, STRUCTURE HARVESTER and online software CLUMPAK were used [39,40]. A threshold membership coefficient of 70% was utilized to assign each individual to a specific group (K), and those that did not meet this criterion were considered as admixed. Additionally, we utilized the software TESS v2.3.1 software to incorporate geographical information into the population structure analysis of wild barley (L3 only) [41]. We also performed principal coordinate analysis (PCoA) in GeneAlex v6.5 to investigate the population structure [42].

### Partitioning of genetic variation explained by environmental and geographical distance in wild barley

For wild barley, the relationships between genetic diversity and geographical and environmental factors were investigated. Climate data for each collecting site were obtained from the Worldclim database 1.4 (www.worldclime.org). The current environmental layers (1950–2000) were downloaded with resolution of 30 arc/sec for 19 bioclimatic variables. For each collection site, the values of 19 bioclimatic variables were extracted and the geographical distribution of wild barley was mapped using ArcGIS 10.3 [43]. To investigate the correlation between geographical, environmental (19 bioclimatic and altitude), and genetic distances, we performed Mantel and partial Mantel tests using the *vegan* package in R v 3.3.2 [44,45] and tested the significance values were tested with 10000 permutations. Pairwise genetic and geographical distance matrices were calculated by GenAlex and for environmental variables standardized Euclidian distance matrix was produced by SPSS v.22 (SPSS Inc., Chicago, IL, USA; http://www.spss.com). We also performed a redundancy analysis (RDA), an alternative method to the Mantel test to examine the relative contribution of environmental and geographic data, and the combination of both in driving genetic structure [44]. RDA is a type of asymmetric canonical analysis based on genetic and environmental matrices and is frequently used by ecologists. Constrained partial RDA determines the relationship between desired variables conditioned on known factors whereas unconstrained partial RDA considers residual variance [46]. In this process, genetic data were used as the response variable, and environmental and geographic data were considered as explanatory variables. Three different models were considered for partitioning variance components of the RDA: (i) a partial model in which the amount of genetic variation is explained by environmental variables conditioning on geographic data, (ii) a partial model in which genetic data is explained by geographic data conditioning on environmental variables, and (iii) a model with all environmental and geographic variables given as explanatory variables. RDA was carried out using the *vegan* package in R [44,45] and significances were determined by 999 permutations.

### Associations between markers and environmental variables in wild barley

We exploited a latent factor mixed model (LFMM) implemented in the LEA package in R [47] to investigate the associations between genetic loci and different environmental variables. LFMM is a recently introduced statistical method based on a hierarchical Bayesian mixed model, which incorporates the residual population structure via latent factors [48,49]. LFMM is robust to the confounding effects of linkage by using hidden factors, when environmental associations are present [48]. For LFMM, we selected a subset of environmental variables that most explained most of environmental variation in wild barley collection sites based on principal component analysis (PCA). For further simplification of biological interpretation, we selected a single environment variable with the strongest loading on each principal component axis (S3 Table). We converted each allele at each locus to binary (0/1) data based on the presence/absence of allele according to the acceptable form of LFMM. The markers with a minor frequency of less than 0.1 were excluded from analysis. Marker-environment associations were determined based on z-scores. The z-scores were estimated based on the Gibbs sampler algorithm by running 10,000 sweeps and using a burn-in length of 5000 for each sweep. The number of latent factors was chosen between 1 and 5 and for any K the program was run five times. To evaluate the inflation of the test statistic, we calculated the genomic inflation factor (λ), based on the approach of Devlin and Roeder [49], in which lambda is used to calculate the adjusted p-values to which determines whether an association is significant. The significant threshold of z-scores was calculated by the Benjamini-Hochberg correction of the adjusted p-values. The markers with z-scores exhibiting a false discovery rate of (q = 0.05) or less were considered significant.

### Identification of candidate genes

For this process, we used the latest barley reference genome available in the IPK database (http://webblast.ipk-gatersleben.de/barley_ibsc) and the Barleymap database (http://floresta.eead.csic.es/barleymap) to determine the candidate genes and annotation of loci surrounding the mapping positions of the SSR markers [50,51]. The protein sequences of putative candidates were then aligned against the *Arabidopsis thaliana* protein sequences by BLASTP to find the probable orthologues using EnsemblPlants (http://plants.ensembl.org).

## Results

### Genetic diversity analysis

Using 40 EST-SSR markers, a total of 375 alleles with fragment sizes between 85 bp (GBM1419) and 363 bp (GBM1002) were obtained across the entire collection (Table 1). The mean number of alleles was 9.375 with the maximum number being 24 alleles for marker GBM1461, and the minimum being 4 alleles for GBM1060 and GBM1404. As shown in Table 1, the PIC values were the lowest for GBM1404, GBM1483, and GBM1060 and highest for marker GBM1461 (PIC value = 0.908), and the mean PIC value was 0.623, indicating high diversity in the collection. The major allele frequency (MAF) parameter varied extensively, ranging from 0.174 for GBM1461 to 0.866 for GBM1404 with a mean value of 0.473. The genetic diversity (GD) values were high, ranging from 0.238 to 0.914; with a mean value of 0.660.

Regarding wild barley a total of 316 alleles with 92 unique alleles were detected with a mean value of 7.9 alleles per locus (Table 1). Markers GBM1007 and GBM1015 had 20 alleles each, while GBM1060 and GBM1404 carried three alleles each. As expected for highly self-pollinating species such as barley, the heterozygosity values were very low for all loci ranging from 0.002 to 0.026, with a mean value of 0.01 across all loci (data not shown). The highest PIC and GD values in wild barley were 0.904 and 0.907, respectively for GBM1007, and the lowest values were 0.151 and 0.156, respectively for GBM1218 (Table 1). For the domesticated genotypes (landraces and cultivars) a total of 283 alleles with a fragment sizes from 85 bp (GBM1419) to 363 bp (GBM1002) were observed, of which 59 alleles were unique. The mean number of alleles in domesticated genotypes was seven, with the maximum number being 20 alleles for GBM1015 and the minimum being 2 alleles for GBM1483. The highest PIC and GD were observed for marker GBM1015 (0.859 and 0.871 respectively) and the lowest values were observed for GBM1404 (0.103 and 0.105 respectively). The MAF values were varied between 0.229 and 0.945 (Table 1). In general, the genetic diversity between the wild and domesticated barley was considerable as domesticated genotypes collected from wider geographical regions (49 countries).

### Population structure analysis of wild and domesticated barley

**Fig 2:**
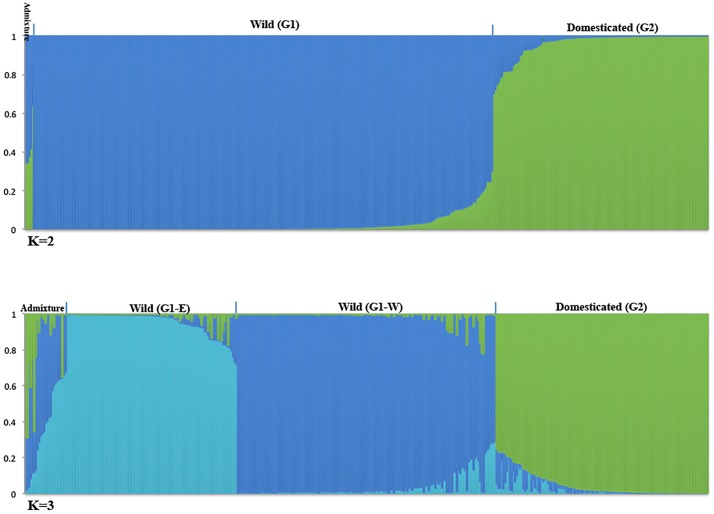
Population structure of wild and domesticated barley populations at K = 2 and K = 3 for the entire collection (L1): Comparison of the STRUCTURE results at K = 2 and K = 3. At K = 2, the blue color represents wild barley and green color represents domesticated barley varieties. At K = 3, the wild barley populations were subdivided into two groups, G1-E and G1-W, which are shown in light and dark blue, respectively. At K = 3, the domesticated varieties (in green) were grouped similarly to what was observed at K = 2. Assignment of individuals to each group was based on their membership coefficient (Q).

**Fig 3:**
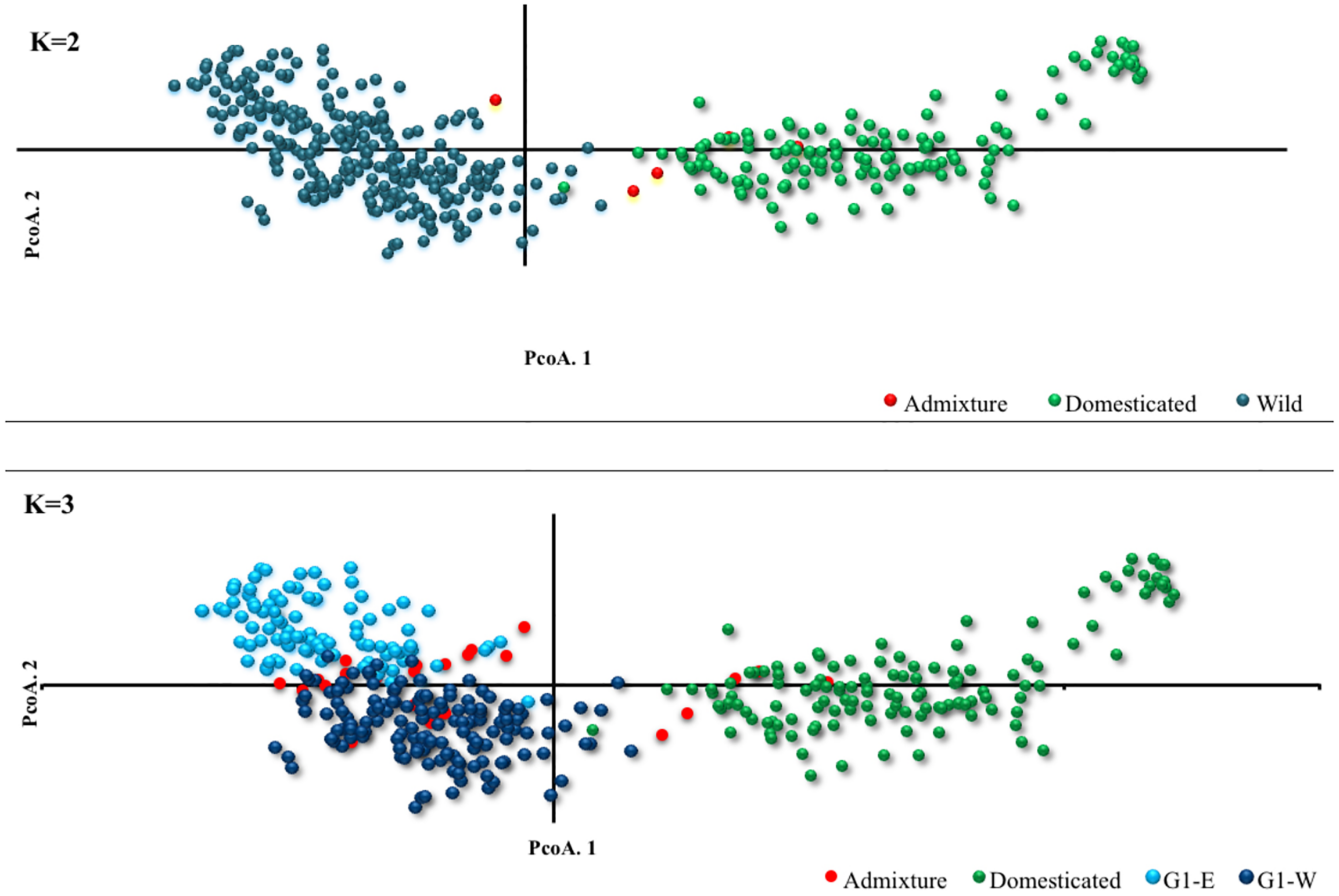
A scatter plot of the first and second PCoA coordinates based on the grouping of barley individuals at K = 2 and K = 3 inferred by STRUCTURE.

At the first level (L1) we performed a population structure analysis across the entire collection (n = 412). To determine the most probable value of K, the LnP(D) values from the STRUCTURE output were plotted against K, and the results showed that these values increased gradually after K = 3. This data suggests three major groups in this collection (S1 Fig). However, the maximum delta (K) from STRUCTURE HARVESTER showed the highest peak at K = 2 (S1 Fig), where wild barley in one group were separated from the domesticated varieties in another group with five individuals being admixed when the 70% criterion was applied (Fig 2). At K = 3, the wild barley genotypes were divided into two groups: the western group (G1-W) located between latitudes from 36.2475 and 37.7058 and longitudes 36.4672 and 38.0122, and eastern group (G1-E) located between latitudes 36.6911 and 38.1536 and longitudes 37.4644 and 41.4564. Applying the same criterion, the domesticated varieties (landraces and cultivars) remained in a single group with 25 admixed individuals (Fig 2). PCoA indicated that the first three coordinates explained 9.96%, 3.53%, and 3.09% of variations for this dataset, respectively. The first PCoA separated wild barley genotypes from the domesticated ones, and the second PCoA separated two wild groups from each other, similar to the results obtained by STRUCTURE at K = 2, and K = 3, respectively (Fig 3).

At the second level (L2) we confined our analysis to genotypes originating only from Turkey (n = 323); i.e., all wild (281 individuals) and 42 domesticated barley varieties. The results were consistent with what we obtained for the entire collection, where at K = 2, the wild barley genotypes were separated from the domesticated ones, and at K = 3, the former were separated into two groups (S2 Fig). Moreover, similar to the case in the entire collection (L1), the membership of wild individuals to their group remained constant with only minor changes.

At the third level (L3), the population structure analysis was confined to wild barley only (n = 281). Similar to the previous results, accessions were clustered in the same groups: i.e., G1-E, and G1-W, with 19 being identified as admixed. This result was also confirmed by the population structure analysis of wild barley using TESS software (S3 Fig). The samples collected from the eastern parts of the study area were clustered into G1-E and those from the western parts were clustered into G1-W. The result also showed that individuals belonging to G1-W were mostly from lower altitudes (150 to 980 meters), while those in G1-E were mainly from higher altitudes (900 to 1140 meters). Interestingly, at all three levels, individuals with admixed membership were observed at middle altitudes (658 to 880 meters).

### Partitioning of genetic variation explained by environment and geography

We used the Mantel and partial Mantel tests to investigate the correlation between eco-geographical variables and genotypic data (Table 2).

**Table 2:** The results of the simple and partial Mantel tests demonstrating the correlation between genetic (Gen), geographic (Geo), and environmental (Env) distances for the wild barley samples from Turkey.

	Mantel test		Partial Mantel test	
	r	p-value	r	p-value
Gen, Geo	0.3876	0.0002	0.2341	0.001
Gen, Env	0.3416	0.0002	0.1321	0.001
Geo, Env	0.6399	0.0002	0.5857	0.001

The highest correlation value was correlated with environmental and geographical distances (r = 0.6399). Both environmental and geographical distances were significantly but not strongly correlated with the genetic distance (r = 0.3416 and r = 0.3876, respectively). Since environmental and geographical distances were correlated, we performed partial Mantel tests to investigate the pure relationships IBE and IBD. The results showed that genetic distance has a reduced but significant correlation with both geographical and environmental distances (r = 0.2341 and r = 0.1321, respectively). However, the correlation between geography and environment did not show a remarkable reduction (r = 0.5857). Based on the information provided by the Mantel test, it can be speculated that geographical and environmental factors have jointly affected the genetic structure of wild barley in the study area, under two groups (G1-E and G1-W), confirming the existence of a spatial structure in the wild gene pool.

We used RDA to further reveal the amount of the genetic variation explained by environmental variables, geographic coordinates and the combination of both (Table 3).

**Table 3:** Partitioning of genetic variation of wild barley accessions using simple and partial redundancy analysis.

Analysis	Factor	Inertia	Percentage (%)	Pr (>F)
Simple RDA	Total	739.55	100	-
	Environmental	79.54	10.75	0.001 ***
	Geographical	10.59	1.432	0.015*
Partial RDA	Total	739.55	100	-
	Environmental	84.37	11.408	0.002***
	Geographical	15.42	2.085	0.001***

Pr(>F) = significant level at ‘***’ 0.001, ‘**’ 0.01, and ‘*’ 0.05.

The results indicated that the variation contributed by environmental variables have played a more important role than the geographic variables. By simple redundancy analysis, the impure proportions of climate and geography on genetic variation were found to be significant (10.75% and 1.43%, respectively). However, the partial RDA was computed in order to separately determine the pure variation contributed by the environmental and geographic variables. Similar to the data obtained above, both factors significantly contributed to the genetic variation of wild barley but the pure environmental portion of genetic variation was higher than the geographic variation (11.41% and 2.08%, respectively).

### Associations between EST-SSR alleles and environmental variables

According to the results presented in S3 Table, the first three principal components explained 88.905% of variations (Eigenvalue >1). In S4 Fig, variables BIO-1 to BIO-11 point to temperature and BIO-12 to BIO-19 indicate to precipitation. The results showed that BIO-19 (precipitation of coldest quarter), BIO-4 (temperature seasonality), and BIO-9 (mean temperature of driest quarter) were the major loadings on PCA1, PCA2, and PCA3, accounting for 38.17%, 36.89%, and 13.83% of total variation, respectively. Hence, we selected these three environmental variables to identify the relationship between allelic variations and environmental variables.

We detected four SSR markers (10%), which possessed at least one allele associated with one bioclimatic variable: GBM1256, GBM1008, GBM1405, and GBM1464 (Table 4). One allele at GBM1256 (354 bp) was associated with BIO-4, four alleles at GBM1008 (171,174,177 and 180 bp) and one at GBM1405 (288 bp) were associated with BIO-9, and two alleles at GBM1464 (126 and 150 bp) were associated with BIO-19. The higher number of associations with BIO-9 reflects the high effect of this environmental variable on the adaptation of wild barley to environmental conditions.

**Table 4:** Associations between EST-SSR marker loci and bioclimatic variables using LFMM based on -Log10 of adjusted p-values, and candidate genes related to these markers based on the latest barley genome sequences in the IPK gene bank.

Bioclimatic Variable	Marker	Chr	Allele (s) in bp	Candidate gene (s)	Protein	Arabidopsis probable ortholog	Role
BIO-4	GBM1256	6H	354	HORVU6Hr1G073690.3	Caleosin-related family protein	AT1G70670 (CLO4)	Negative regulator in ABA signaling. Play role in drought and high salinity stresses (Kim et al. 2011; Khalil et al. 2014)
				HORVU6Hr1G073710.1	Calmodulin binding protein-like	AT1G73805 (SARD1)	Encodes SAR Deficient 1 (SARD1), a key regulator for ICS1 (Isochorismate Synthase 1) induction and salicylic acid (SA) synthesis (Truman et al. 2013)
BIO-9	GBM1008	6H	171, 174, 177, 180	HORVU6Hr1G082350.5	Chaperone protein DnaJ (HSP40)	AT3G14200	Heat shock proteins (Hsps). Play role against biotic abiotic stress (Wang et al. 2004; Park and Seo 2015)
	GBM1405	3H	288	HORVU3Hr1G082000.2	Adenine nucleotide alpha hydrolases-like superfamily protein	AT1G44760	Role in flooding tolerance (Ayyappan et al. 2017)
				HORVU3Hr1G082070.5	serine hydroxymethyltransferase 7	**AT1G36370 (SHMT7)**	minimize the production of ROS in chloroplasts and mitigate oxidative damage (Moreno et al. 2004)
BIO-19	GBM1464	7H	126*, 150	HORVU7Hr1G029650.4	Transcriptional corepressor SEUSS	**AT5G62090** (SLK2)	Forming an important LUH-SLK2 complex to play role against abiotic stress (Shrestha et al. 2014)
				HORVU7Hr1G029640.1	calmodulin like 23	**AT1G66400**	regulate plant responses to environmental stresses (Zeng et al. 2015)

*: Found only in the wild gene pool

### Identification of candidate genes

Using the IPK database we performed an *in silico* analysis of the associated markers to detect underlying candidate genes (Table 4). The first marker associated with BIO-4 (GBM1256) was corresponded to the gene model encoding Caleosin-related family protein (CLO) which is located 54Kb upstream of the gene similar to *SYSTEMIC ACQUIRED RESISTANCE DEFICIENT1* (*SARD1*) in *Arabidopsis thaliana*, an important member of the calmodulin binding protein-like gene family. Of two markers associated with BIO-9, one (GBM1008) was found in the region corresponding to the gene encoding chaperone protein DnaJ (HSP40) and the other associated marker (GBM1405) appeared to underlie the gene encoding a protein belonging to the adenine nucleotide alpha hydrolases-like superfamily, located 306 Kb downstream of another interesting gene similar to *serine hydroxymethyltransferase 7* (*SHMT7*) in *A*. *thaliana*. Finally, the marker associated with BIO-19 (GBM1464) was found in a gene region similar to *SLK2* which encodes transcriptional corepressor SEUSS in *A*. *thaliana*, and was located 179Kb downstream of the gene similar to *CML23* encoding calmodulin-like proteins in *A*. *thaliana*.

## Discussion

### Genetic diversity and population structure in wild barley

In the present study, the genetic diversity analysis revealed a high number of alleles (375 in total) across the entire collection of wild and domesticated barley. This is comparable to the results previously reported using the same marker set: a worldwide collection of 1,485 barley landraces genotyped with 42 markers with 372 alleles [17], a worldwide collection of 224 spring barley accessions genotyped by 45 markers with 228 alleles [35], and 185 domesticated and 38 wild barley genotypes with 356 alleles were detected by 45 markers [52]. Of the 375 alleles revealed, 283 were present in domesticated and 316 in the wild barley genotypes (Table 1). It is expected that wild barley was more diverse than domesticated barley due to the higher selection pressure on the latter during the domestication process. The higher number of alleles within the wild gene pool might be explained by adapting strategy against various environmental factors, which finally led to localized adaptation. The PIC and genetic diversity values were slightly higher for the domesticated varieties (Table 1), possibly due, in part, to the limited geographical distribution of wild barley and selective breeding of domesticated barley for specific traits.

The analysis of the population structure across the entire collection resulted in two major clusters of wild barley exist in southeastern Anatolia, indicating a selection pressure during evolution [20]. The few numbers of admixtures among the accessions was likely associated with a low gene flow from the adjacent regions. Moreover, we found that the wild barley samples collected in the study area could be categorized into two groups: eastern (G1-E) and western (G1-W). When assigning wild accessions into their structure inferred clusters, we noticed that G1-W group was assigned to lower altitudes less than (<980 meters) and G1-E contained those from the higher altitudes (>900 meters). Populations from highlands are often exposed to more precipitation, which is presumably another reason for local adaptation and why higher altitudes can be an obstacle to gene flow. These results are in accordance with previous reports on barley accessions in Turkey, indicating two main clusters [20]. We compared the clustering membership of wild genotypes that were common between this study and in Jakob et al. [20] assigned the in which wild barley individuals to three clusters: “Western cluster” encompassing individuals collected from Israel, Greece, Lebanon, Jordan, Syria, and southern Turkey; “Turkish cluster” comprising individuals from southeast Turkey, and “Eastern cluster” consisting of individuals from an are covering Turkey, Iran, Uzbekistan and Tajikistan. In the current study, the genotypes grouped under G1-W were the same as those in the “Turkish cluster”[20], and those clustered into G1-E corresponding to the “Western cluster”[20].

### The joint role of eco-geographical factors in wild barley genetic makeup

We performed Mantel tests and redundancy analysis as a complementary approach to detect the complex multivariate relationships. As stated above, both geographic and environmental (bioclimatic and altitude) factors were important in explaining different aspects of the population structure of wild barley in Turkey. The significant correlations between geographical and genetic distances as well as between environmental and genetic distances indicate a spatial distribution, influenced by natural selection. Using the Mantel test, a strong significant correlation (r = 0.6399) was observed between geographical and environmental distances. The partial Mantel test showed that genetic distance still has a significant, but reduced correlation with environment or geography (Table 2). Furthermore, according to the redundancy analysis, among the geographical factors, longitude had a higher contribution than latitude (data not shown). Turkey is located in wider range of longitudes (It lies between latitudes 35° and 43° N, and longitudes 25° and 45° E), therefore, it is expected that longitude would be more determinative than latitude in differentiating between groups. Nevo et al. [53] stated that longitude and altitude were more effective than latitude in forming genetic structure in the northern parts of the Fertile Crescent, indicating that specific geographical parameters strongly alter the environmental factors. It is clear that the genetic makeup of wild barley in the study area has been affected by the contribution of both geography and environment. However, environmental factors could be more influential as reflected by their greater proportion in explaining total genetic variance through RDA. As was also explained in the next section, results obtained from principal components analysis on environmental parameters of the collection sites further confirmed the important roles of joint contribution of precipitation and temperature on the genetic differentiation of wild barley groupd inferred from STRUCURE (S3 Table). Several studies have confirmed that environmental factors play significant role in adaptation of wild barley and other plant species [54–58]. Using the partial Mantel test, Hübner et al [59] found that flowering time and some phenotypic traits were significantly correlated with temperature and rainfall gradients. In another previous study, the allozyme diversity of wild barley populations from Turkey displayed sharp geographic differentiation over short distances and some allele frequencies of wild barley were ecologically predictable through the combinations of temperature and humidity variables [53]. It was also noted that the spatial patterns of genetic variation of *H*. *spontaneum* in Turkey was not only common, but also at least partly adaptive [53]. Thus, spatial distribution cannot be solely explained by a simple isolation by distance (IBD) model and requires more factors that influence the observed genetic structure, such as biotic and abiotic stresses [23]. Similar results have been reported for wild barley accessions from Israel based on SSR markers, demonstrating the major role of ecological factors as selective forces in the adaptation of wild barley to this part of the Fertile Crescent [22–24].

Abebe et al. [60] found that climate factors accounted for 40% of the variation explained, whereas the geographical factors were considered less important in the genetic diversity of barley landraces from Ethiopia. The authors also observed a significant correlation between different altitude classes with the population structure but a weak correlation with geographical factors. On the contrary, the population structure of wild barley populations from Jordan was associated with IBD at a large scale but no correspondence between climate and genetic structure [6]. This means that global climatic data cannot solely explain the existing genetic diversity in Jordan [6]. Finally, our results indicate that the spatial distribution of wild barley in the study area is not randomly but is associated with both geographical and environmental factors, which together resulted in local adaptation. Jakob et al. [20] stated that variables describing temperature and precipitation regimes during extreme quarters appeared to restrict the potential distribution. Furthermore, the authors suggested that the current distributional predictions were fairly good representations of the taxon's extant geographical distribution and this was also represented by the relatively high contribution of environmental predictors describing temperature and precipitation.

### Association of alleles with environmental variables

The associations between EST-SSR alleles and environmental factors identified eight associated alleles from four different loci associated with both temperature and precipitation variables. Of the three environmental variables, BIO-9 (mean temperature of driest quarter) had the maximum number of associated alleles, followed by BIO-19 (precipitation of coldest quarter) and BIO-4 (temperature seasonality), with two and one correlated alleles, respectively. The distribution of wild barley is shown on the map of Turkey for the selected bioclimatic variables (S5 Fig). The wild barley populations clustered in the G1-E group has been mostly collected from sites with lower BIO-9 values compared to the individuals to G1-Wgroup. In relations to BIO-4, nearly all the G1-W group members were located in areas with higher values than those in the G1-E group, indicating that wild barley genotypes from the western parts (G1-W) may be exposed to higher seasonal variation in temperature (S5 Fig). In contrast, the wild barleypopulations belonging to G1-E were located in the sites with higher values of BIO-19. These environmental variables appear to play a significant role in differentiating populations through selection pressure and local adaptation.

In this study, GBM1008, GBM1256 (on chromosome 6H), and GBM1405 (on chromosome 3H) were associated with temperature factors while GBM1464 (on chromosome 7H) was associated with the precipitation variable. Among the eight alleles associated with climatic variables, one allele (GBM1464-126) was not present in the domesticated gene pool. In their investigation of 94 samples of *Hordeum chilense* from Chile, Castillo, et al. [57] detected 12 outlier loci associated with eco-geographical factors including GBM1008 and GBM1464, which is also supported by our results. Drought stress has a complex nature and often interacts with other factors such as temperature extremes. Recently, Sayed et al. [61] reported several quantitative trait loci associated with root architecture traits in barley. One locus (QSRR30.S42-6H) located on chromosome 6H was linked to marker bPb-6477, which is in close proximity (4.4 cM) to GBM1008 (associated with BIO-9 in our study). These results indicate that chromosomes 3H, 6H and 7H contain key genomic regions associated with abiotic adaptation in barley.

### Identification of candidate genes

The loci associated with environmental variables were further searched in the database for putative candidate genes. We found that these associated markers were located in the regions containing candidates involved in various abiotic stresses (Table 4). The associated marker GBM1256 is located on chromosome 6H in a region where two other important candidates are exist: one encoding a caleosine-related protein (CLO) and another encoding a calmodulin binding protein (SARD1). Caleosins are lipid-budy associated proteins that Ca^2+^ -dependent peroxygenase activity and have been shown to play an important role in response to drought and salinity stresses [62,63]. Another important gene encodes a protein similar to Systemic Acquired Resistance Defficient1 (SARD1) in *A*. *thaliana*, a positive regulator of plant immunity that promotes the production of salycilic acid (SA) and induces a wide range of SA-dependent and SA-independent genes [64]. The marker GBM1008 on chromosome 6H was corresponded to a gene that encodes chaperone DnaJ, also called a J-domain-containing protein (J-protein) a member of heat shock proteins (HSP40). A well-studied aspect of thermotolerance in plants is related to the accumulation of HSPs in response to heat and various environmental stresses [65]. Five major families of HSPs exist in plants: HSP100, HSP90, HSP70, HSP60, and small HSPs (sHPS) [65,66]. These proteins are involved in stabilizing and resolubilizing denatured proteins during heat stress. Another associated marker was GBM1405 on chromosome 3H in a region corresponding to the gene encoding adenine nucleotide alpha hydrolases-like protein and close to the gene encoding a protein similar to serine hydroxymethyltransferase 7 (SHMT7) in *A*. *thaliana*. In switchgrass, adenine nucleotide alpha hydrolases have been reported to be involved in flooding tolerance and that are implicated in leaf senescence [67]. SHMTs are antioxidant enzymes and are shown to be involved in the photorespiratory pathway and play an important role in scavenging the accumulation of reactive oxygen species (ROS) and mitigating oxidative damage [68]. The marker GBM1464 associated with BIO-19 on chromosome 7H was in a region corresponding to the gene encoding transcriptional corepressor SEUSS, an ortholog of Arabidopsis *SLK2*. A recent study reported that SLK1 and SLK2 interact with LEUNIG_HOMOLOG (LUH) forming SLK1-LUH and SLK2-LUH co-repressor [69]. These complexes regulate genes that are disadvantageous to the abiotic stress tolerance [69]. For example, plants can employ mechanisms to delay important growth stages that are sensitive to abiotic stress tolerance. The LUH-SLK1 and LUH-SLK2 complexes could repress the genes that are involved in the transition of growth phase [69]. Another interesting gene surrounding the region was the one encoding Calmodulin like protein (CML). CMLs are major Ca^2+^ sensors that play critical roles in interpreting crypted Ca^2+^ signals and regulate various types of proteins, most of them directly or indirectly regulating plant responses to environmental stresses [70]. In conclusion we propose that the identified candidates have high potential of adaptation to climate variables and could be regarded as potential targets in response to environmental changes. Finally, the results suggest the high potential value of wild barley from southern Anatolia as resource for future breeding programs.

## Supporting information

S1 Fig The results of the STRUCTURE analysis for 412 wild and domesticated barley. (A), the mean logarithm of probability values, LnP(D) against the number of predefined clusters (K); (B), Magnitude of delta K vs. K values obtained from STRUCTURE HARVESTER.
(PDF)

S2 Fig The population structure of the wild and domesticated barley from Turkey inferred by STRUCTURE. At K = 2, blue colour represents wild barley and green colour represents the domesticated group in L2. At K = 3, the wild barley were subdivided into two groups G1-E (light blue) and G1-W (dark blue). Assignment of individuals to each group was based on their membership coefficient (Q).
(PDF)

S3 Fig The population structure of wild barley inferred by TESS in southeastern Anatolia. At K = 3, wild barley from the western part (G1-W) was separated from those from the eastern part (G1-E), shown in dark blue and light blue, respectively in L3.
(PDF)

S4 Fig Coding for 19 bioclimatic variables according to the WorldClim database. All temperature variables are based on degrees in Celsius except for Bio3, which defined as the percentage and quantifies how large the day-to-night temperatures oscillate relative to the summer-to-winter (annual) oscillations. All precipitation variables are based on millimeter, except for Bio15 and is expressed as a percentage which is a measure of the variation in monthly precipitation totals over the course of the year. Bio15 is the ratio of the standard deviation of the monthly total precipitation to the mean monthly total precipitation (also known as the coefficient of variation).
(PDF)

S5 Fig The distribution of wild barley genotypes on a map of Turkey according to the associated bioclimatic variables by LFMM at K = 2. BIO4: temperature seasonality (The amount of temperature variation over a given year (or averaged years) based on the standard deviation (variation) of monthly temperature averages. The values are based on original program Arc Map Language (AML^®^) available at http://www.worldclim.org/bioclim which multiplies the result by 100 (SD * 100), which was designed to preserve significant digits. This variable groups genotypes based on standard variation so that those which have the maximum standard deviation highlighted in red color and lower amounts are in other colors. For instance, red group shows genotypes which their standard variation is between 9200 to 9800. In fact, this group have experienced much thermal variation during years. BIO9: mean temperature of driest quarter (This index approximates mean temperatures that prevail during the driest quarter. This map groups barley based on the temperature differences in various colors, for example blue color contains genotypes which have endured less temperatures than others (between 23.5 to 24.5 Degrees Celsius). Note that according to values from WorldClim database, temperature data are in °C * 10. BIO19: precipitation of coldest quarter (This index approximates total precipitation that prevails during the coldest quarter. Genotypes located in the same color indicated with the same rainfall amounts in a unique group, for instance, red color illustrates the group in which genotypes located that have tolerated minimum rainfall (180 to 210 mm).
(PDF)

S1 Table An overview of accessions including names, status, population name, collection sites, geographical coordinates and membership to the STRUCTURE inferred groups. (XLSX)

S2 Table Sequence information for all 40 EST-SSR markers used in this study (35). (PDF)

S3 Table The loading values of environmental variables on PCA axes. (PDF)
