# Supplementary figures and images for: Geographical and environmental determinants of the genetic structure of wild barley in southeastern Anatolia
Source: PLoS One. 2018 Feb 8;13(2):e0192386. doi: 10.1371/journal.pone.0192386 (PMC5805283; doi:10.1371/journal.pone.0192386)

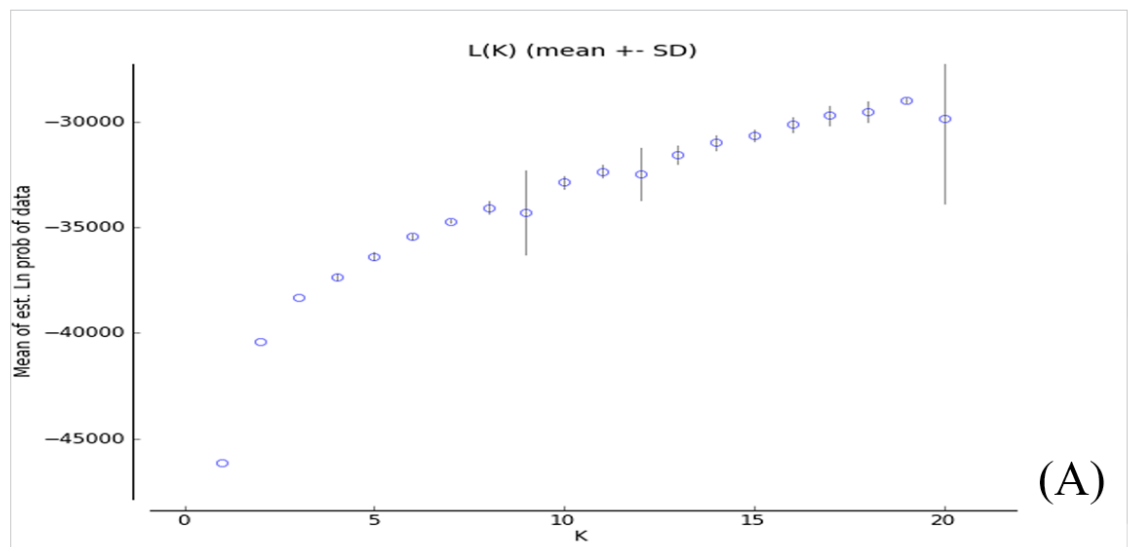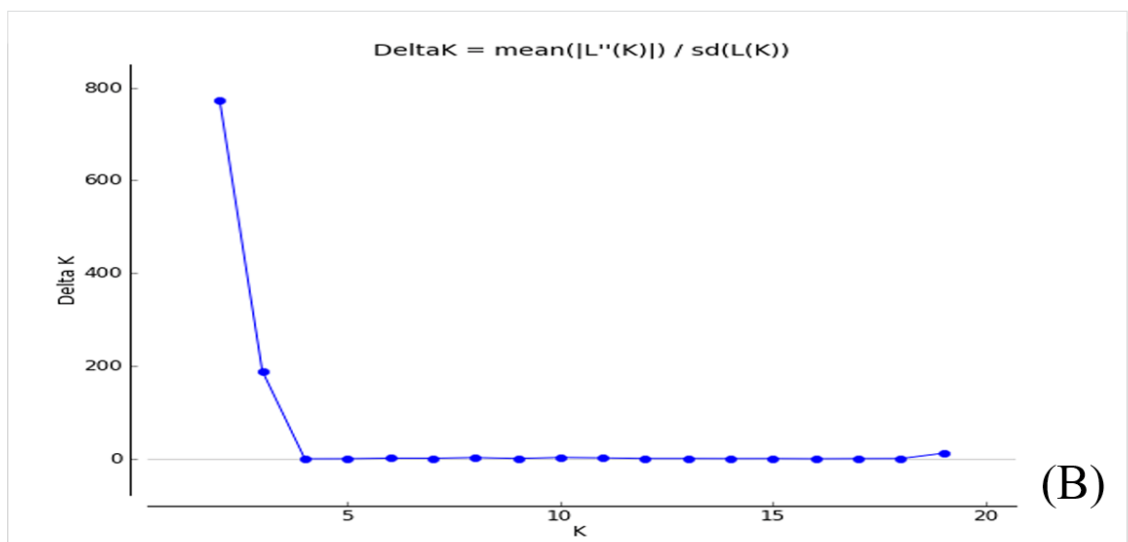

**S1 Fig.**

Supplement: S1 Fig — (A), the mean logarithm of probability values, LnP(D) against the number of predefined clusters (K); (B), Magnitude of delta K vs. K values obtained from STRUCTURE HARVESTER. (PDF) [file pone.0192386.s001.pdf]

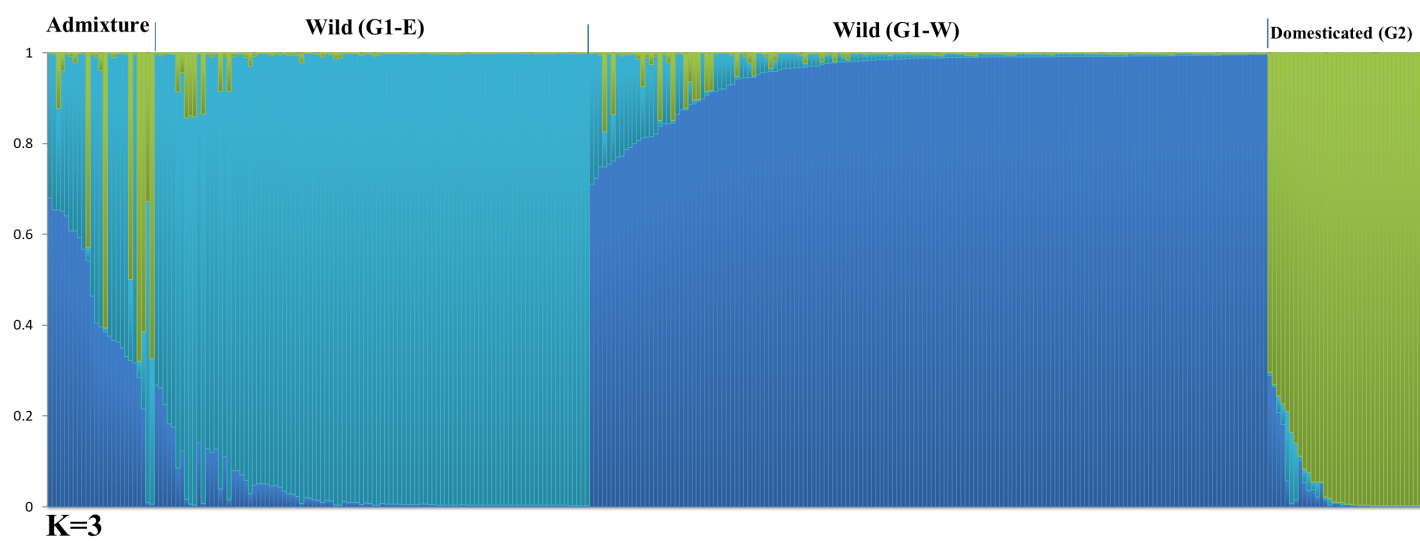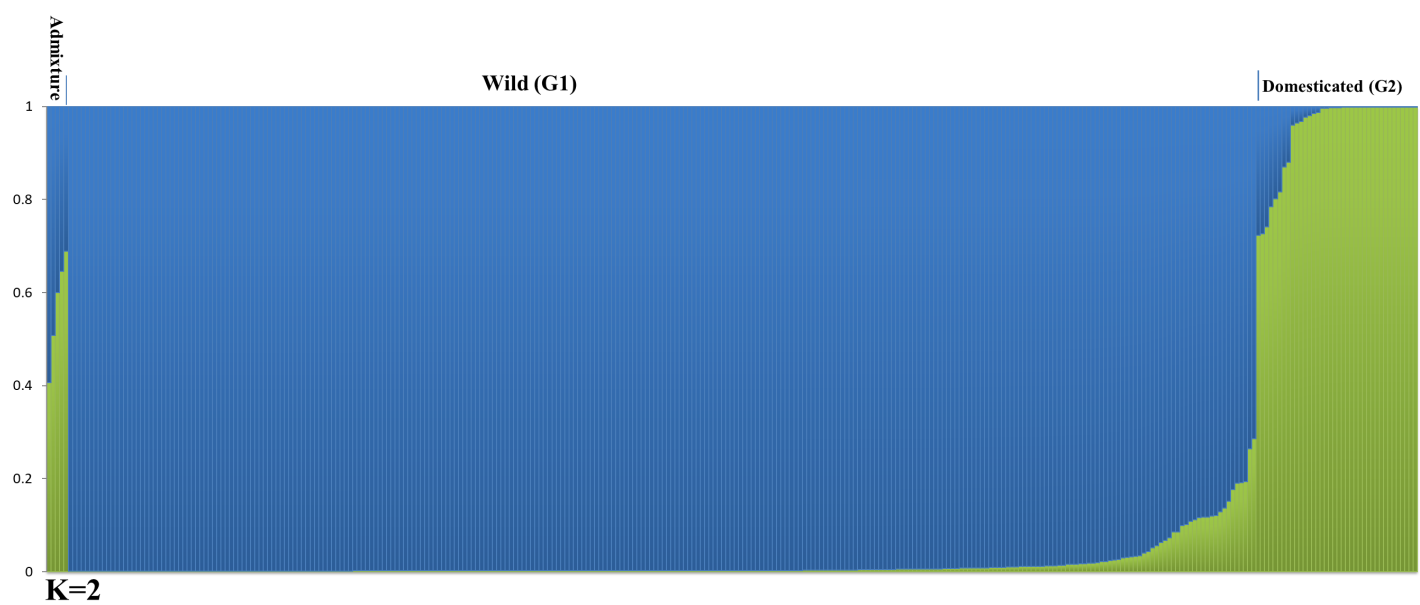

**S2 Fig.**

Supplement: S2 Fig — At K = 2, blue colour represents wild barley and green colour represents the domesticated group in L2. At K = 3, the wild barley were subdivided into two groups G1-E (light blue) and G1-W (dark blue). Assignment of individuals to each group was based on their membership coefficient (Q). (PDF) [file pone.0192386.s002.pdf]

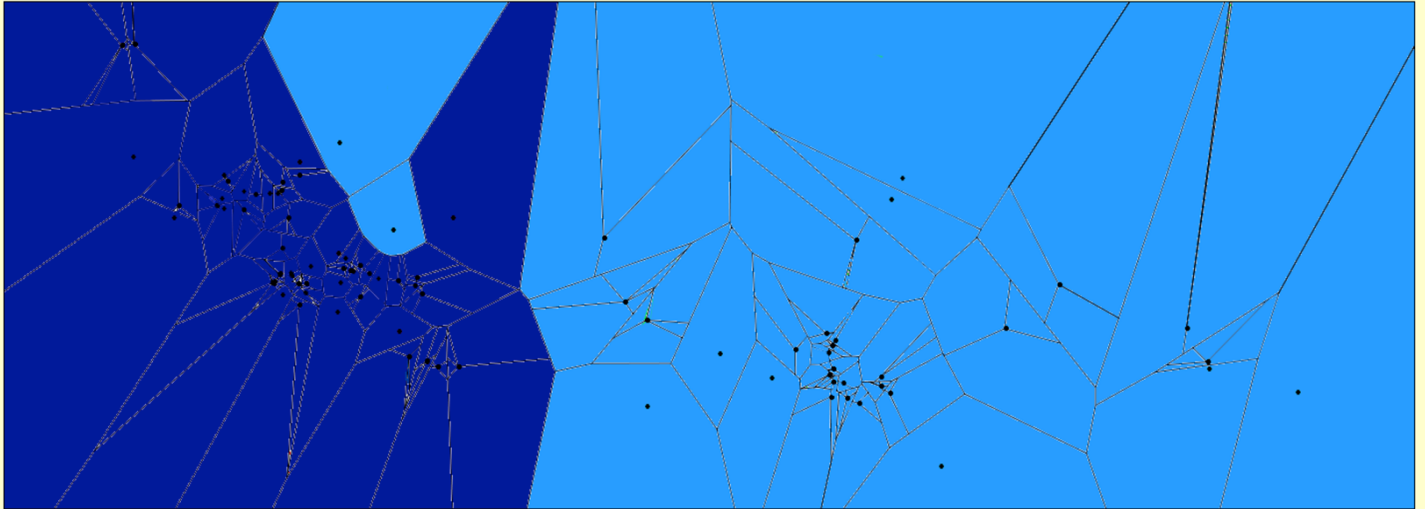

S3 Fig.

Supplement: S3 Fig — At K = 3, wild barley from the western part (G1-W) was separated from those from the eastern part (G1-E), shown in dark blue and light blue, respectively in L3. (PDF) [file pone.0192386.s003.pdf]

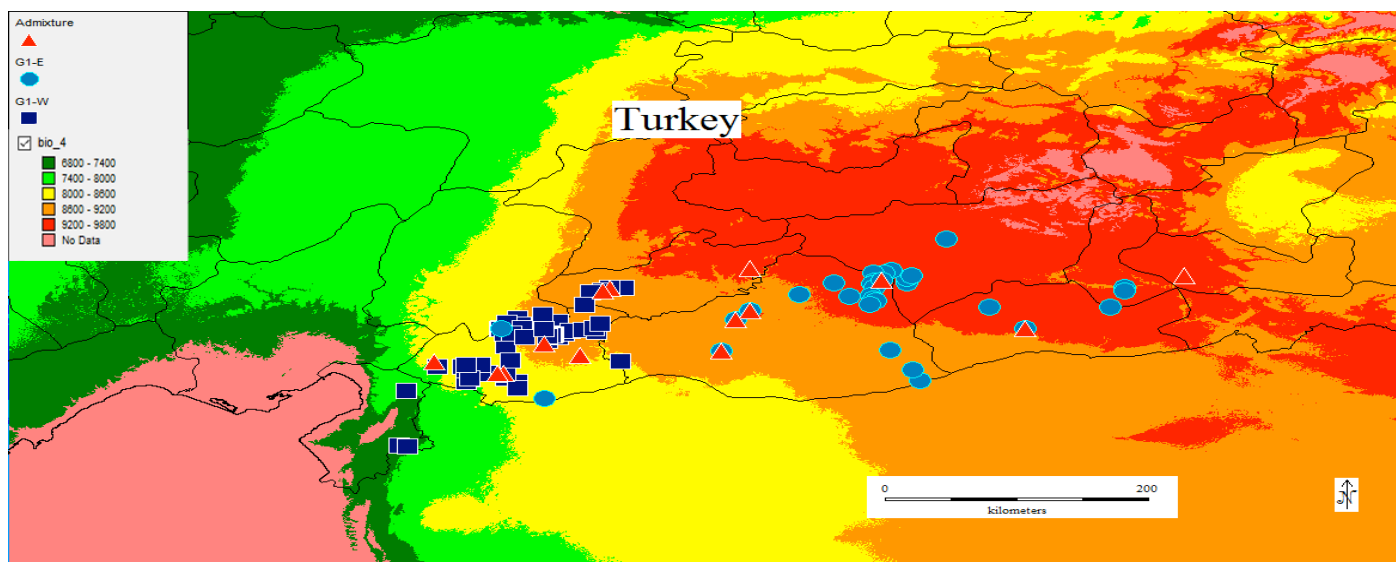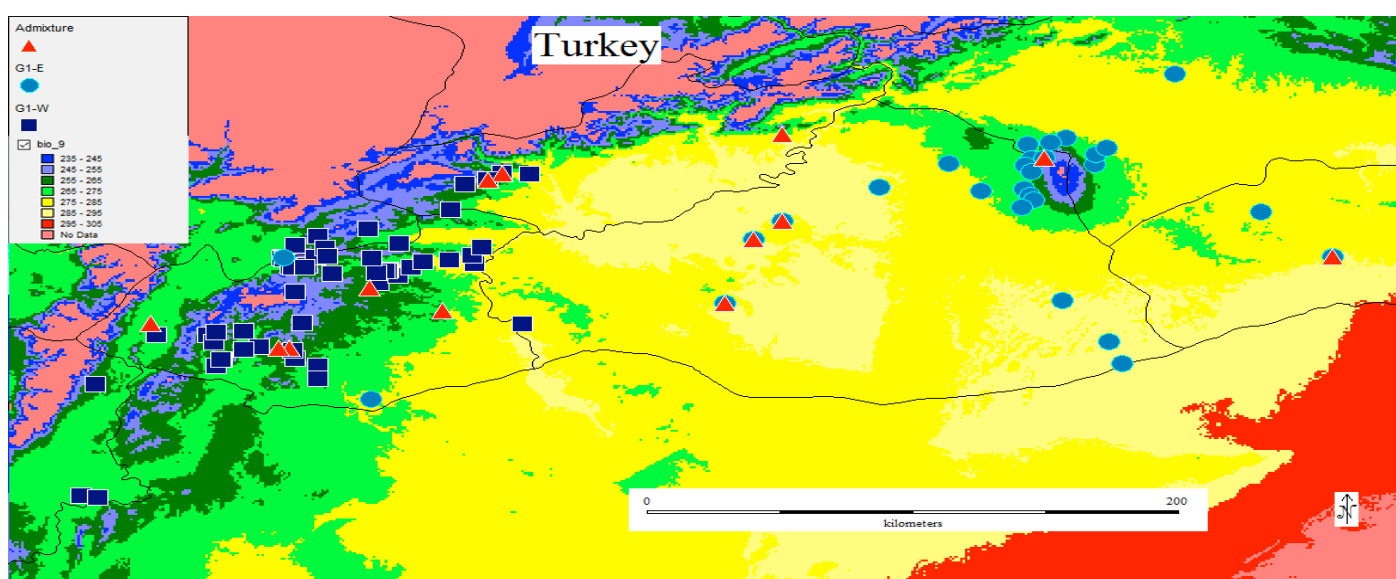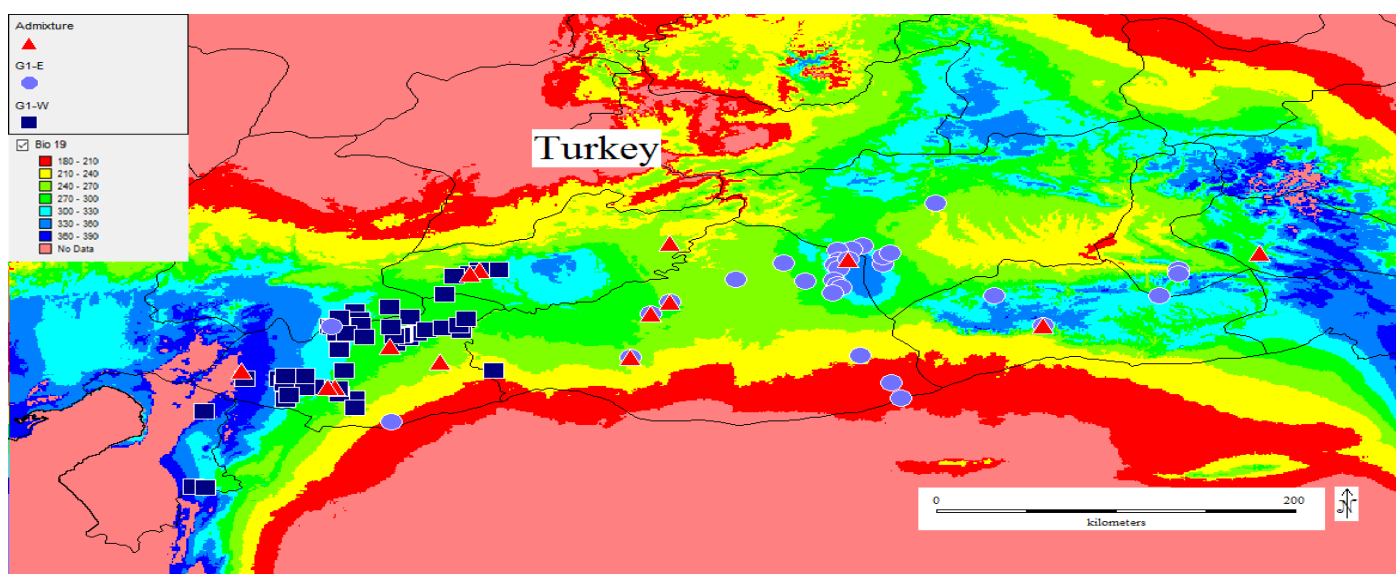

S5 Fig.

Supplement: S5 Fig — BIO4: temperature seasonality (The amount of temperature variation over a given year (or averaged years) based on the standard deviation (variation) of monthly temperature averages. The values are based on original program Arc Map Language (AML®) available at http://www.worldclim.org/bioclim which multiplies the result by 100 (SD * 100), which was designed to preserve significant digits. This variable groups genotypes based on standard variation so that those which have the maximum standard deviation highlighted in red color and lower amounts are in other colors. For instance, red group shows genotypes which their standard variation is between 9200 to 9800. In fact, this group have experienced much thermal variation during years. BIO9: mean temperature of driest quarter (This index approximates mean temperatures that prevail during the driest quarter. This map groups barley based on the temperature differences in various colors, for example blue color contains genotypes which have endured less temperatures than others (between 23.5 to 24.5 Degrees Celsius). Note that according to values from WorldClim database, temperature data are in °C * 10. BIO19: precipitation of coldest quarter (This index approximates total precipitation that prevails during the coldest quarter. Genotypes located in the same color indicated with the same rainfall amounts in a unique group, for instance, red color illustrates the group in which genotypes located that have tolerated minimum rainfall (180 to 210 mm). (PDF) [file pone.0192386.s005.pdf]
