# Supplementary material for: Geographical and environmental determinants of the genetic structure of wild barley in southeastern Anatolia
Source: PLoS One. 2018 Feb 8;13(2):e0192386. doi: 10.1371/journal.pone.0192386 (PMC5805283; doi:10.1371/journal.pone.0192386)
Supplement: S4 Fig — All temperature variables are based on degrees in Celsius except for Bio3, which defined as the percentage and quantifies how large the day-to-night temperatures oscillate relative to the summer-to-winter (annual) oscillations. All precipitation variables are based on millimeter, except for Bio15 and is expressed as a percentage which is a measure of the variation in monthly precipitation totals over the course of the year. Bio15 is the ratio of the standard deviation of the monthly total precipitation to the mean monthly total precipitation (also known as the coefficient of variation). (PDF) [file pone.0192386.s004.pdf]

Variables		Details
Bio1	Temperature	Annual mean temperature
Bio2		Mean diurnal range (mean monthly min. temp.—mean monthly max. temp.)
Bio3		Isothermality (Bio2/Bio7)
Bio4	Temperature	Temperature seasonality (SD)
Bio5		Maximum temperature of the warmest month
Bio6		Minimum temperature of the coldest month
Bio7		Annual temperature range (Bio5-Bio6)
Bio8		Mean temperature of the wettest quarter
Bio9		Mean temperature of the driest quarter
Bio10		Mean temperature of the warmest quarter
Bio11	Temperature	Mean temperature of the coldest quarter
Bio12	Precipitation	Annual precipitation
Bio13		Precipitation of the wettest month
Bio14	Precipitation	Precipitation of the driest month
Bio15		Precipitation seasonality (coefficient of variation)
Bio16		Precipitation of the wettest quarter
Bio17		Precipitation of the driest quarter
Bio18		Precipitation of warmest quarter
Bio19	Precipitation	Precipitation of coldest quarter

S4 Fig.
