# Supplementary material for: Geographical and environmental determinants of the genetic structure of wild barley in southeastern Anatolia
Source: PLoS One. 2018 Feb 8;13(2):e0192386. doi: 10.1371/journal.pone.0192386 (PMC5805283; doi:10.1371/journal.pone.0192386)
Supplement: S2 Table — (PDF) [file pone.0192386.s007.pdf]

S2 Table.

NAME	EST_ID	F_primer	FORWARD_PRIMER_SEQ	R_primer	REVERSE_PRIMER_SEQ
**GBM1218**	HI01I18w	ESSR1083	TGACTGGCACTCACAACACA	ESSR1084	AGCGCTTCCATCCTTGACTA
**GBM1047**	HY04G20u	ESSR305	GCACGACGGTAACAGGTTTT	ESSR306	CGGAGTACCTGAGCCTTCTG
**GBM1419**	HY01A18V	ESSR1485	CGTCACGCCACTCACCTC	ESSR1486	CTTGAAGTCGGAACCCATGT
**GBM1516**	HX05K11r	GBM1516_f	CCCTCTCCTTTCCCTATCGT	GBM1516_r	GTGGGGTTGATGTTCCTGTT
**GBM1064**	HW09C19u	ESSR583	TAAAGAATTGCTGGCTGGCT	ESSR584	GAAGGTGTTTTTGTGCCCAT
**GBM1060**	HW05A06u	ESSR561	CGGGGGAGACTGGATTTTAT	ESSR562	CTCCTCCAGAGGAAGCTCGT
**GBM1035**	HK05M02r	ESSR199	ACTGAAGAGTGAAAACGGCG	ESSR200	GGCGTACCACCAGTACATCC
**GBM1075**	HW02M04T	ESSR659	AACAAGAAGCCCACCATCAC	ESSR660	ATCCAAGGTGGCAGTCAATC
**GBM1007**	HW06D21u	ESSR41	GTTCCGGTTTCTTTCGGTC	ESSR42	GTTAGCTATGGTGCGGTGTG
**GBM1404**	HU03I13u	ESSR1455	CAAGCCAGCAAACAAACAAA	ESSR1456	GGGAGTACGTGTTGCTCCAT
**GBM1280**	HA04E19u	ESSR1207	CTTCTTCTTCTTGTTGGGCG	ESSR1208	AAGGGATCAGTTTGGTTCCC
**GBM1461**	HD03P05u	GBM1461_f	AAACCATGCATTCTTCAGAGA	GBM1461_r	TTTAGACCGACCCGATGAAG
**GBM1020**	HK06H24r	ESSR131	CAGACAAACAGCAACCCAGA	ESSR132	GATTCTTTACGGCGAATGGA
**GBM1026**	HW09G19u	ESSR149	AGGCTCTTCACCGTCTCGTA	ESSR150	CATCACCGCTTGGTTCTACA
**GBM1015**	HW04P19u	ESSR115	TTGTTGGAACATACAAACATGC	ESSR116	GTTCCGTGTGAATTAGCGGT
**GBM1031**	HY03N03T	ESSR177	CAGTTGGCTTCTACCCCAAA	ESSR178	GCTACGACCCACAACAACAA
**GBM1029**	HK05M17u	ESSR165	AGAACCAACAAGCCCTTCCT	ESSR166	AGAAGACGCGATCCTCTTGA
**GBM1176**	HU01M19w	ESSR999	TATACATCAGCGGGCCTTTT	ESSR1000	CTCCAACCTCGCAAAGAGTC
**GBM1464**	HD13P04r	GBM1464_f	ATAGCCGTGCTCTTGCTCAT	GBM1464_r	CAAGACCACCATTTGCATTG
**GBM1002**	HW07P19V	ESSR19	TCCAAACAAACAGACTCGCA	ESSR20	CCCAGTAGCTCTTCTGCACC
**GBM1013**	HW08M23V	ESSR95	TCCCTGATCCATGTTTTTCC	ESSR96	GATGCCAAGTTCTTCTTCGC
**GBM1033**	HY02O24u	ESSR193	AGCAAATGTTGAGCAACGG	ESSR194	ACTTCATAGGGCGGAGGTCT
**GBM1061**	HW09H12V	ESSR563	CTGAGATCCCGAACACCACT	ESSR564	TTGTCGTCCACGCTCACTAC
**GBM1110**	HI01I17w	ESSR867	CACCGAAGAAAATCCACCAT	ESSR868	CACGCAAGCTTAGAACCTCC

**GBM1208**	HX01M04T	ESSR1063	CTACCGAGCTCCTCCTCCTC	ESSR1064	GGCCTCCTTCTTGTCGTAGA
**GBM1405**	HX02N04u	ESSR1457	TACACGCACTGAAAAGACGG	ESSR1458	CTCGCTGCTGAGTTTGTCTG
**GBM1221**	HW02L22u	ESSR1089	ACCAGCAATCCAAGTTACGG	ESSR1090	TGCCTTGGTCTTGGTGTGTA
**GBM1483**	HI11G22r	GBM1483_f	CAGTGATATGGACTACGGCG	GBM1483_r	CTTGTTCTCCACCTCGAAGC
**GBM1003**	HW04D20V	ESSR25	GCCGACCAGATCATCAGATT	ESSR26	TCGAAGATTCAGTAGCGACG
**GBM1256**	HA01K04r	ESSR1159	GCCTCGATCTGTGGAAAGAA	ESSR1160	GTCTCGGAGAAGGTGACGAT
**GBM1018**	HY03J01T	ESSR125	CTGCACACACACACCCTAGC	ESSR126	CCGTACTTTGAGAAGAGGCG
**GBM1334**	HV01J13T	ESSR1315	CCTCTTCCGAATCCATCAAA	ESSR1316	GAGTTGGGGACTTTGCTGAC
**GBM1363**	HM03O19r	ESSR1373	AAGAAGGCCAAGCTGAGACA	ESSR1374	CCTCGATAGGCTCTGTCCTG
**GBM1008**	HY08G04u	ESSR47	CAGCAATGGACACTGAATCAA	ESSR48	GTCGAAGGCTATGTAGGCCC
**GBM1212**	HX01P07w	ESSR1071	TGTTGCAAGAAGCAAGGATG	ESSR1072	GCGCTTACTCTCTCGTCGTC
**GBM1413**	HV04M04u	ESSR1473	GGGTGATTTCCCAGGTTTTT	ESSR1474	TTGAGAAAACCACACCCACA
**GBM1063**	HW05L08u	ESSR579	CGGTACCAATTCTTTGATTACCA	ESSR580	TCCAGTCTTCGCATCATCTG
**GBM1459**	HD03E20u	GBM1459_f	AACACATCCATACTTCCCCG	GBM1459_r	AGCTGAATAAATGCCCATGC
**GBM1501**	HS08I08u	GBM1501_f	TGTGGACAGTCAAACACGCT	GBM1501_r	AAGGAGCACCTGAAAAGCAA
**GBM1323**	HZ01J22r	ESSR1293	GCTCTCCAGGGTTCGTTTC	ESSR1294	CACCGTCTTGCAGTTGAGAC
