# Supplementary material for: Geographical and environmental determinants of the genetic structure of wild barley in southeastern Anatolia
Source: PLoS One. 2018 Feb 8;13(2):e0192386. doi: 10.1371/journal.pone.0192386 (PMC5805283; doi:10.1371/journal.pone.0192386)
Supplement: S3 Table — (PDF) [file pone.0192386.s008.pdf]

S3 Table.

Environmental variable	PCA 1	PCA 2	PCA 3
Altitude	0.144	-0.222	-0.016
BIO1	-0.214	0.266	0.211
BIO2	-0.232	-0.063	-0.350
BIO3	-0.092	0.290	-0.296
BIO4	-0.029	-0.354	0.139
BIO5	-0.305	-0.138	0.143
BIO6	-0.119	0.340	0.104
BIO7	-0.099	-0.343	0.010
BIO8	-0.163	0.278	0.174
BIO9	-0.270	0.009	0.391
BIO10	-0.291	0.013	0.338
BIO11	-0.141	0.334	0.085
BIO12	0.271	-0.066	0.355
BIO13	0.312	0.121	0.159
BIO14	0.259	0.012	-0.179
BIO15	-0.093	0.202	-0.380
BIO16	0.310	0.113	0.181
BIO17	0.239	0.252	-0.017
BIO18	0.227	0.148	0.022
BIO19	0.313	0.112	0.167
